# Insights on Quorum-Quenching Properties of *Lysinibacillus fusiformis* Strain RB21, a Malaysian Municipal Solid-Waste Landfill Soil Isolate, via Complete Genome Sequence Analysis

**DOI:** 10.1128/genomeA.00409-15

**Published:** 2015-05-07

**Authors:** Delicia Yong, Robson Ee, Yan-Lue Lim, Chien-Yi Chang, Wai-Fong Yin, Kok-Gan Chan

**Affiliations:** a Division of Genetics and Molecular Biology, Institute of Biological Sciences, Faculty of Science, University of Malaya, Kuala Lumpur, Malaysia; b Interdisciplinary Computing and Complex BioSystems (ICOS) Research Group, School of Computing Science, Newcastle University, Claremont Tower, Newcastle upon Tyne, United Kingdom; c The Centre for Bacterial Cell Biology, Medical School, Newcastle University, Newcastle upon Tyne, United Kingdom

## Abstract

*Lysinibacillus fusiformis* strain RB21 is a quorum-quenching bacterium that is able to degrade quorum-sensing signaling molecules. Here, we present the first complete genome sequence of *L. fusiformis* strain RB21*.* The finished genome is 4.8 Mbp in size, and the quorum-quenching gene was identified.

## GENOME ANNOUNCEMENT

*Lysinibacillus fusiformis* strain RB21 was isolated from a nonoperational municipal solid waste landfill site in Malaysia. Equipped with quorum-quenching (QQ) ability, *L. fusiformis* strain RB21 is able to degrade quorum-sensing (QS) signaling molecules, namely, *N*-acyl-homoserine lactone (AHL). As QS regulates the expression of various genes that are vital for bacterial pathogenicity (1), QQ is garnering attention as a potential alternative for the treatment of bacterial infections (2). In this study, we sequenced and here report the first complete genome sequence of *L. fusiformis*, with the objectives of studying this bacterium comprehensively and characterizing its QQ gene.

The MasterPure DNA purification kit (Epicentre) was used to extract the genomic DNA according to the manufacturer’s instructions. The Qubit 2.0 fluorometer (Invitrogen, USA) and NanoDrop spectrophotometer (Thermo Fisher Scientiﬁc, USA) were used to determine the concentration and purity of the extracted genomic DNA, respectively. A 10-kb SMRTbell template library was then constructed and sequenced using the PacBio RSII single-molecule real-time (SMRT) sequencing platform (Pacific Biosciences, USA) (3, 4). From the sequencing output, a total of 124,500 reads with a mean read length of 4,993 bp were generated. The filtered insert reads were subsequently assembled into two scaffolds using the Hierarchical Genome Assembly Process (HGAP) *de novo* assembly workflow (version 2) (Pacific Biosciences) (5).

Gene prediction and genome annotation were performed using RAST (6), Prokka (7), Integrated Microbial Genomes-Expert Review (IMG-ER) (8), RNAmmer (9), tRNAscan-SE (10), and the NCBI Prokaryotic Genome Annotation Pipeline (PGAP).

The genome of *L. fusiformis* strain RB21 comprises a circular chromosomal genome (4,843,789 bp, 37.62% G+C content, and 104.3-fold coverage) and a circular plasmid (17,280 bp, 53.67% G+C content, and 673.45-fold coverage). Annotation analysis revealed the presence of 4,900 genes, of which 4,721 genes were protein-coding genes, whereas another 179 genes were RNA genes (42 rRNA and 107 tRNA genes). In addition, proteins encoded by 3,018 genes were assigned to 21 Clusters of Orthologous Groups (COGs). A QQ gene, *N*-acyl-homoserine lactonase, was also found in the genome. The complete genome sequence of *L. fusiformis* strain RB21 provides the first genetic evidence of its QQ activity.

### Nucleotide sequence accession numbers.

This complete genome sequence has been deposited in DDBJ/ENA/GenBank under the accession numbers CP010820 (chromosome) and CP010821 (plasmid). The versions described in this paper are the first versions, CP010820.1 and CP010821.1.
